# Cardioprotective effects and pharmacokinetic properties of a controlled release formulation of a novel hydrogen sulfide donor in rats with acute myocardial infarction

**DOI:** 10.1042/BSR20140185

**Published:** 2015-06-22

**Authors:** Ba Hieu Tran, Chengrong Huang, Qiuyan Zhang, Xu Liu, Shizhou Lin, Hongrui Liu, Shujun Wang, Yi Zhun Zhu

**Affiliations:** * School of Pharmacy, Fudan University, Shanghai, Shanghai, China; † School of Pharmacy, Shenyang Pharmaceutical University, Shenyang, Liaoning, China; ‡ School of Pharmacy & Institutes of Biomedical Sciences, Fudan University, Shanghai, Shanghai, China

**Keywords:** acute myocardial infarction, anti-inflammation, antioxidant, cardioprotection, hydrogen sulfide

## Abstract

We previously reported that *S*-propargyl-cysteine (SPRC) exerts cardioprotective effects by elevating H_2_S levels via the CSE/H_2_S pathway. In the present study, we investigated the cardioprotective effects and pharmacokinetic properties of a controlled release formulation of SPRC (CR-SPRC) in an *in vivo* rat model of myocardial infarction (MI). Rats were randomly assigned to seven groups that were pre-treated with CR-SPRC daily for 7 days prior to ligation of the left anterior descending coronary artery to induce MI. Cardiac function and infarct size were determined after MI, and we examined the activity of antioxidant enzymes, expression of anti-inflammation proteins and hydrogen sulfide levels. Mixed-mode, reversed-phase and cation-exchange HPLC–MS/MS were used to compare the pharmacokinetic properties of CR-SPRC and SPRC. CR-SPRC significantly reduced infarct size and creatine kinase (CK) and lactate dehydrogenase (LDH) leakage and it preserved cardiac function during MI. CR-SPRC displayed antioxidant properties, preserving glutathione (GSH), catalase (CAT) and superoxide dismutase (SOD) levels whereas reducing malondialdehyde (MDA) levels. Moreover, CR-SPRC significantly reduced the protein levels of inflammatory biomarkers (phospho-NF-κB p65/NF-κB p65, TNF-α) and increased cystathionine-γ-lyase (CSE) and Iκ-Bα protein levels. CR-SPRC had better pharmacokinetic properties than SPRC, with a reduced concentration peak (*C*_max_), prolonged time to reach peak concentration (*T*_max_), prolonged mean residence time (MRT_inf_) and increased AUC_0–*t*_. CR-SPRC showed protective effects against MI via the CSE/H_2_S pathway and demonstrated better cardioprotective effects than SPRC by prolonging the release of endogenous H_2_S.

## Abstract (precis)

The present study confirms that CR-SPRC is stable, effective and is thus an alternative candidate for hydrogen sulfide-mediated long-term prevention of cardiovascular diseases.

## INTRODUCTION

Despite some recent improvements in diagnosis and treatment, cardiovascular diseases remain the leading cause of death worldwide and myocardial infarction (MI) is among the most prevalent ischemic heart diseases. Extensive evidence has established the importance of oxidative stress and inflammation in the pathogenesis of MI. MI initiates oxidative stress and an intense inflammatory response that promotes myocardial apoptosis, cell death, ventricular remodelling and cardiac dysfunction [1–3]. Therefore, antioxidative and anti-inflammatory responses may be vital for preventing apoptosis, salvaging the myocardium, preserving heart function and reversing cardiac remodelling.

Previously, we reported the cardioprotective effects of *S*-propargyl-cysteine (SPRC), an exogenous donor of hydrogen sulfide, *in vitro* and *in vivo.* SPRC exerted cardioprotective effects against hypoxia and myocardial and vascular endothelial cell injury in various inflammatory conditions *in vitro* [4] and it reduced the extent of MI, myocardial ischemia/reperfusion injury and heart failure *in vivo*. Moreover, SPRC exerted cardioprotection by increasing the activity of a pyridoxal-5-phosphate-dependent enzyme, cystathionine-γ-lyase (CSE) and plasma H_2_S concentrations [5,6].

Recently, H_2_S has been identified as a third endogenous gas signalling molecule along with nitric oxide and carbon monoxide, playing an important function in many pathophysiological process of the mammal. In the cardiovascular system, H_2_S executes the physiological functions of vasorelaxation, angiogenesis and ion channels [7,8]. H_2_S plays a critical role in protection against oxidative stress, apoptosis and inflammation in the cardiovascular diseases including hypoxia, hypertension, atherosclerosis, myocardial ischemia and heart failure [9–16]. Moreover, H_2_S also has many protective effect against the disease of nervous system, gastrointestinal system, respiratory system and immune system [17–19]. There are several mechanism directing the protective effects of H_2_S. First, H_2_S may activate several ion channel including K_ATP_ channels, T-type calcium channels and ankyrin transient receptor potential channel (TRPA1). Second, H_2_S is involved in the activation of Akt signalling pathways and simultaneously enhances vascular endothelial growth factor (VEGF) production and VEGFR2 phosphorylation. Third, H_2_S may cause an inhibition of NF-κB signalling pathway and cytochrome oxidase leading to protection against ischemia-associated tissue damage. Finally, H_2_S may also directly enhance antioxidant enzymes superoxide dismutase (SOD) and glutathione peroxidase (GSH-Px), which act as a scavenger of reactive oxygen and nitrogen species [9,19].

In the heart, H_2_S is produced by CSE using L-cysteine as a substrate. SPRC, which is structurally similar to cysteine, promotes the activity of CSE, increasing H_2_S production [4,5]. Thus, SPRC represents a promising target for treating MI. However, because SPRC contains sulfur amino acids and polar molecules, it is dissolved, absorbed and eliminated very quickly; therefore, it must be repeatedly administered to maintain an effective concentration. These properties have limited its clinical application because stable and sustainable effects cannot be obtained. To solve these problems, we selected Eudragit (R) RS30D as a carrier and employed a solid dispersion technique to develop a controlled-release formulation of SPRC (CR-SPRC). The present study aimed to investigate the pharmacokinetic properties of CR-SPRC and its cardioprotective effects in MI rats.

## METHODS

Animal care and experimental procedures were approved by the Department of Animal Care and Use Committee of Fudan University, and all experiments were performed in accordance with the Guide for the Care and Use of Laboratory Animals of the Ministry of National Health and Family Planning of the People's Republic of China. All surgeries were performed under anaesthesia with 7% chloral hydrate (35 mg/kg i.p.) to ameliorate animal suffering.

### Drugs

Propagylglycine (PAG, a CSE inhibitor) was purchased from Sigma–Aldrich. Captopril was purchased from Sine Pharmaceutical Co.. SPRC was synthesized as previously described [6]. Professor Wang Shujun provided CR-SPRC (School of Pharmacy, Shenyang Pharmaceutical University) and it was synthesized using a solid dispersion technique, as described previously [20]. SPRC and CR-SPRC were suspended in 0.5% sodium carboxymethylcellulose (CMC-Na) for treatment.

### Animal model and experimental design

Male SPF Sprague–Dawley rats (8–10 weeks old, weighing 200–220 g) were purchased from Sippr-bk Experimental Animal Center. The animals were kept under standard conditions at 24±1°C, 55–60% humidity, with a 12-h light–dark cycle and they were allowed free access to food and tap water throughout the experiment. The rats were randomly divided into seven groups: Sham treated with vehicle (CMC-Na) (*n*=11), MI treated with vehicle (CMC-Na) (*n*=22), MI treated with CR-SPRC (30 mg/kg per day) (*n*=22), MI treated with SPRC (30 mg/kg per day) (*n* =22), MI treated with PAG (15 mg/kg per day) (*n*=22), MI treated with CR-SPRC + PAG (30 mg/kg per day and 15 mg/kg per day, respectively) (*n*=22) and MI treated with captopril (30 mg/kg per day) (*n*=22). Body weight was measured daily to adjust the drug dosages. PAG was injected intraperitoneally, and all other drugs were administered intragastrically. The drugs were administered daily for 7 days, and the MI model was induced on day 7, as described previously [21]. Briefly, rats were anesthetized and maintained under anaesthesia by intraperitoneal injection 7% choral hydrate (35 mg/kg i.p.). The chest was opened between the third and fourth rib using a small animal chest expander, and the left descending anterior coronary was ligated 2–3 mm from its origin using a 5.0 silk thread. The success of the model was verified by the observation of rapid discolouration in the ischemic area from pink to grey (anterior ventricular wall and the apex) and elevation of the ST segment immediately after ligation. The chest was closed in layers, and the skin was sterilized with povidone iodine. The animals remained on the warm pad and were allow to recover under care to consciousness in a stable condition. After surgery, the drug treatment was continued for 2 days, and animal death was recorded every day to determine mortality. Rats that died within 48 h of the operation were excluded from the following experiments. The animals were humanely killed on day 2 after surgery by overdose with 7% chloral hydrate (70 mg/kg i.p.). Subsequently, blood samples (2 ml) were collected to measure H_2_S concentrations. MI biomarker like creatine kinase (CK), lactate dehydrogenase (LDH), catalase (CAT) and oxidation biomarker like SOD activities and GSH and malondialdehyde (MDA) concentrations were detected using a diagnostic kit (NJBI). The heart was removed from the animals immediately after death, washed in ice-cold saline and stored at −80°C for subsequent experimental assays.

### Assessment of cardiac function by echocardiography

An echocardiographic study was performed at day 5 after the MI procedure using a Vevo770 system (Visual Sonics Inc.) with 17.5-MHz transducers as described previously [20]. The rats were anesthetized with 7% chloral hydrate (35 mg/kg i.p; *n* =6 per group) and placed in the supine position. A cardiologist who was blinded to the drug treatment evaluated the cardiac function of the rats. The left ventricular internal dimension in systole (LVIDs), left ventricular internal dimension in diastole (LVIDd), left ventricular anterior wall in systole (LVAWs), left ventricular anterior wall in diastole (LVAWd), left ventricular posterior wall in systole (LVPWs), left ventricular posterior wall in diastole (LVPWd), left ventricular volume in systole (LVs), left ventricular volume in diastole (LVd), ejection fraction (EF) and fractional shortening (FS) were measured using the M-mode tracings of the high-resolution electrocardiograph system.

### Measurement of infarct size

Infarct size was determined as described previously [6]. Briefly, the entire heart was cut into six horizontal slices (*n*=6 per group) and incubated in 0.1% 2,3,5-triphenyltetrazolium chloride (TTC) solution at 37°C for 30 min. Then, the slices were washed three times with saline and fixed in 4% formalin for 30 min. Finally, the slices were placed on a glass slide and photographed using a digital camera. The infarct was measured using ImageJ software (NIH), and the size is expressed as a percentage of the infarct area with respect to the entire left ventricular area.

### Measurement of H_2_S concentration

The concentration of H_2_S in the supernatant was determined as described previously [6]. Briefly, 75 μl of plasma (*n*=6 per group), 425 μl of distilled water and 133 μl of *N*,*N*-dimethyl-phenylenediamine sulfate (NNDPD, 20 μM) in 7.2 M HCl and FeCl_3_ (30 μM; 133 μl) in 1.2 M HCl were successively added to a microtube containing zinc acetate (1% w/v; 250 μl). Finally, trichloroacetic acid (TCA, 10% w/v; 250 μl) was added to precipitate any protein. The resulting solution was centrifuged at 24,000 *g* for 5 min at 4°C. The optical absorbance of the supernatant at 670 nm was determined using a 96-well microplate reader (Tecan Systems Inc.).

### Assessments of protein levels by western blot analysis

Frozen left ventricular tissue (*n*=6 per group) was cut into small pieces (50 mg per group) and homogenized using a rotor-stator homogenizer in 1.0 ml of ice-cold RIPA buffer (Beyotime Biotechnology). Subsequently, the homogenates were centrifuged at 13,000 *g* at 0°C for 10 min, and the supernatant was collected. Protein concentrations were determined using an enhanced BCA protein assay kit (Beyotime Biotechnology). After boiling with loading buffer (Beyotime Biotechnology) at 100°C for 5 min, proteins were loaded and separated on a 12% acrylamide SDS/PAGE gel. After electrophoresis, the separated proteins were transferred electrophoretically to a PVDF membrane (Millipore Corporation). The membranes were incubated in blocking buffer (5% non-fat dry milk in TBST (TBS containing 0.05% Tween)) for 1 h to block nonspecific binding, followed by incubation with the following primary antibodies: polyclonal rabbit anti-CSE (1:500), polyclonal rabbit anti-NF-κB p65 (1:1000), polyclonal rabbit anti-phospho-NF-κB p65 (1:500) (Ser536), polyclonal rabbit anti-Iκ-Bα (1:1000), polyclonal rabbit anti-TNF-α (1:1000) (Cell Signaling Technology, Inc.) and GAPDH (1:1000) (Santa Cruz Biotechnology). After incubation with HRP-conjugated goat anti-rabbit IgG (ICL Lab) as a secondary antibody for 1 h at room temperature, proteins were visualized using enhanced chemo-luminescence with a camera-based imaging system (Alpha Innotech). The density of the signals was quantified using AlphaEase software.

### Pharmacokinetic application

Rats were fasted for 12 h before and 3 h after dosing, and they were allowed free access to water. Twenty-four rats were randomly grouped in four major groups (*n*=6 per group): normal treated with CR-SPRC, normal treated with SPRC, MI treated with CR-SPRC and MI treated with SPRC. All rats received 30 mg/kg SPRC in 0.5% CMC-Na via intragastric (i.g.) administration. Blood samples (200 μl) were collected by tail vein puncture at 0.5, 1, 2, 4, 6, 8, 10, 12, 14, 24 and 30 h after administration. SPRC plasma concentrations were determined using mixed-mode, reversed-phase and cation-exchange HPLC–MS/MS methods, as previously described [22]. DAS 2.0 software (Mathematical Pharmacology Professional Committee of China) was used to calculate pharmacokinetic parameters. The AUC_0−_*_t_* was estimated using the linear trapezoidal method. *T*_max_ was analysed using a nonparametric test (Kruskal–Wallis test).

### Statistical analysis

Data are expressed as the mean ± S.D. Differences between multiple groups were examined using one-way analysis of variance (ANOVA). Differences between two groups were analysed using two-tailed Student's *t*test. The significance of mortality data was determined using chi-squared test. Statistical significance was defined as *P*< 0.05, and all analyses were performed using SPSS 12.0.

## RESULTS

### Mortality and infarct size

Mortality was determined according to the percentage of rats that died within 48 h after surgery with respect to the entire population. Mortality was significantly lower in the CR-SPRC and SPRC groups than in the MI group (9.1% and 13.6% compared with 40.9%, *P*<0.05, respectively). Mortality was significantly lower in the CR-SPRC group than in the PAG group (9.1% compared with 36.4% *P*< 0.05). There was no difference in mortality between the MI group and the captopril group.

TTC staining demonstrated that CR-SPRC and SPRC significantly reduced the infarct size in the left ventricle compared with the MI group (18.5±4.8% and 21.8±2.1% compared with 35.8±4.4%; *P*< 0.01, respectively) (Figure 1). The infarct was also smaller in the CR-SPRC and SPRC groups than in the captopril group (25.5±2.3%; *P*< 0.01 and *P*< 0.05, respectively).

**Figure 1:**
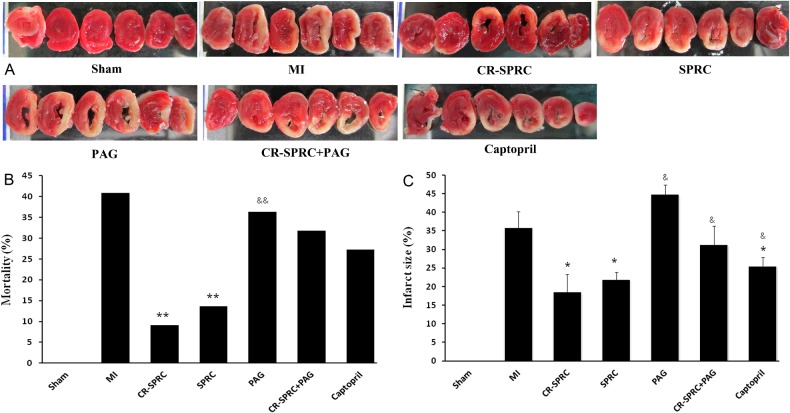
Effect of CR-SPRC on myocardial infarct size and mortality Mortality (**B**) and myocardial infarct size (**A**, **C**). Data are presented as the mean ± S.D. (*n*=6). *P< 0.01 compared with vehicle; **P< 0.05 compared with vehicle; ^&^*P*< 0.01 compared with CR-SPRC; ^&&^*P*< 0.05 compared with CR-SPRC.

### Lactate dehydrogenase activity, creatine kinase activity and plasma lipid peroxidation level

As show in Figure 2, CK activity was significantly higher in the MI and PAG groups than in the CR-SPRC group. CK activity was also higher in the SPRC group than in the CR-SPRC group (0.20±0.02 units/ml, *P*< 0.05). Similarly, LDH levels were remarkably higher in the MI and PAG groups than in the CR-SPRC group. However, these effects were abolished in the CR-SPRC plus PAG treatment group, and there was no significant difference in CK and LDH levels between the MI and SPRC groups. Moreover, lipid peroxidation (as determined by MDA levels) was lower in rats treated with CR-SPRC and SPRC than in the MI and PAG groups. Notably, the MDA level was remarkably lower in the CR-SPRC group than in the SPRC group (*P*< 0.01, Figure 3).

**Figure 2:**
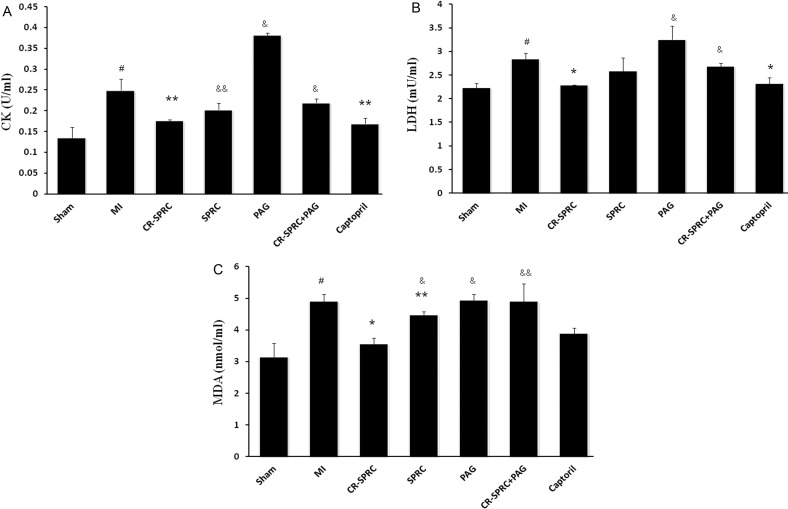
Effect of CR-SPRC on CK, LDH and MDA levels Plasma CK (**A**), LDH (**B**) activity and MDA (**C**) concentrations. Data are presented as the mean ± S.D. (*n*=6). ^#^*P*< 0.01 compared with Sham; *P< 0.01 compared with vehicle; **P< 0.05 compared with vehicle; ^&^*P*< 0.01 compared with CR-SPRC; ^&&^*P*< 0.05 compared with CR-SPRC. All experiments repeated at least three times.

**Figure 3:**
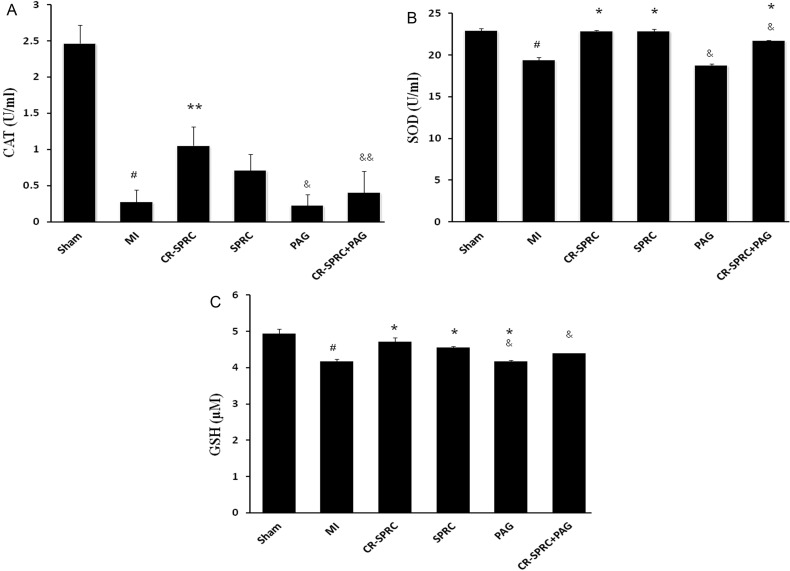
Effect of CR-SPRC on CAT, SOD and GSH levels Plasma CAT (**A**), SOD (**B**) and GSH (**C**) concentrations. Data are presented as the mean ± S.D. (*n*=6). ^#^*P*< 0.01 compared with Sham; *P< 0.01 compared with vehicle; **P< 0.05 compared with vehicle; ^&^*P*< 0.01 compared with CR-SPRC; ^&&^*P*< 0.05 compared with CR-SPRC. All experiments repeated at least three times.

### Catalase and superoxide dismutase activity and glutathione levels

The levels of three plasma antioxidant biomarkers, i.e., CAT activity, SOD activity and GSH levels, were significantly higher in the CR-SPRC treatment group than in the MI group. SOD activity and GSH levels were significantly higher in the CR-SPRC plus PAG treatment than in the MI group. However, the increase in CAT activity was abolished in rats treated with CR-SPRC plus PAG. There was no difference in the levels of these three plasma antioxidant markers between the CR-SPRC treatment group and SPRC group (Figure 3).

### H_2_S levels in plasma

Compared with the MI vehicle group, the plasma H_2_S concentration in the CR-SPRC and SPRC treated groups was increased by 1.8-fold and 1.4-fold, respectively (24.19±1.88 μM and 18.92±1.50 compared with 13.56±0.44 μM, *P*< 0.01). The CR-SPRC-treated group had a higher H_2_S level than the SPRC-treated group (1.3-fold, *P*< 0.05). The PAG-treated group had the lowest H_2_S concentration (9.60±0.96 μM, *P*< 0.01), and the plasma H_2_S concentrations of the CR-SPRC + PAG-treated groups were significantly lower than those of the CR-SPRC-treated groups (17.47±0.23 μM, *P*< 0.01, Figure 4).

**Figure 4:**
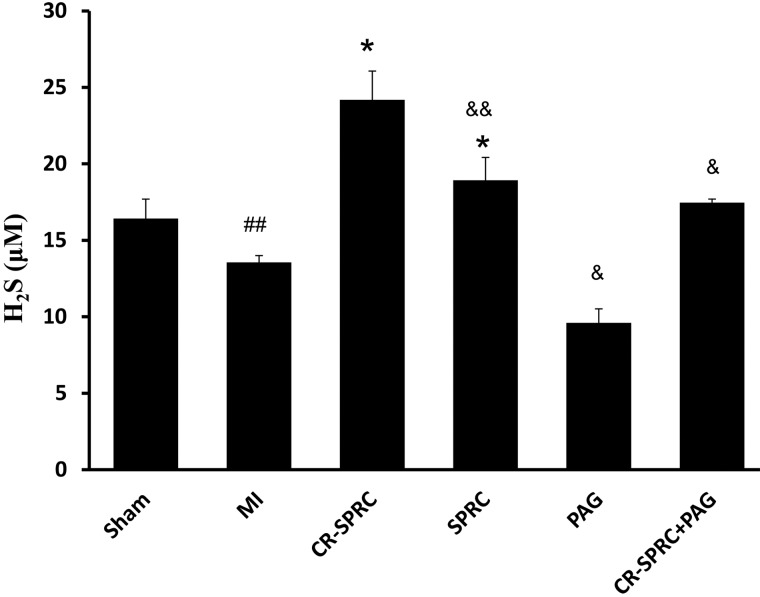
Effect of CR-SPRC on H_2_S concentrations Plasma H_2_S concentrations. Data are presented as the mean ± S.D. (*n*=6). ^##^*P*< 0.05 compared with Sham; *P< 0.01 compared with vehicle; ^&^*P*< 0.01 compared with CR-SPRC; ^&&^*P*< 0.05 compared with CR-SPRC.

### Anti-inflammatory effects of CR-SPRC

CSE protein expression was 1.3-fold (*P*< 0.01) and 1.2-fold (*P*< 0.05) higher in the CR-SPRC- and SPRC-treated groups, respectively, than in the MI vehicle group. Iκ-Bα protein expression was 1.2-fold (*P*< 0.05) and 1.4-fold (*P*< 0.05) higher in the CR-SPRC- and SPRC-treated groups, respectively, than in the MI vehicle group. TNF-α, phospho-NF-κB p65/NF-κB and p65 protein expression was 1.3-fold (*P*< 0.05) lower in the CR-SPRC group than in the MI vehicle group. There was no difference in TNF-α, phospho-NF-κB p65/NF-κB and p65 protein expression between the SPRC-treated and MI vehicle groups (Figure 5).

**Figure 5:**
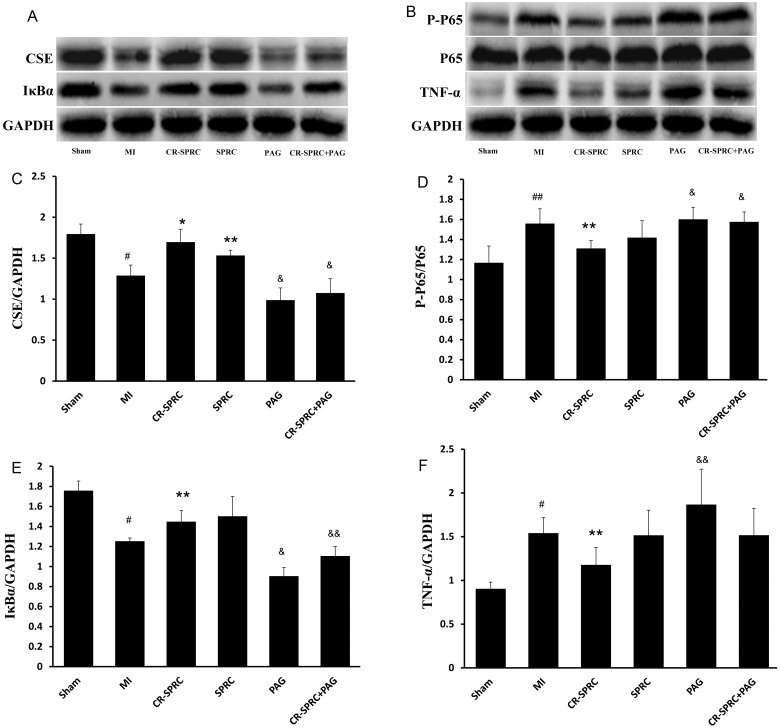
Effect of CR-SPRC on protein expression Protein expression of CSE (**A**, **C**) and inflammatory markers (**A**, **B**, **D**–**F**) in the left ventricle. Data are presented as the mean ± S.D. (*n*=6). ^#^*P*< 0.01 compared with Sham, ^##^*P*< 0.05 compared with Sham; *P< 0.01 compared with vehicle; **P< 0.05 compared with vehicle; ^&^*P*< 0.01 compared with CR-SPRC; ^&&^*P*< 0.05 compared with CR-SPRC. All experiments repeated at least three times.

### Echocardiography results

On day 5 after MI induction, EF, FS and LVAWs were notably lower in the MI group than in the Sham group (43.38% ± 9.04% compared with 75.62% ± 5.82%; 22.26% ± 5.49% compared with 45.13% ± 5.29% and 1.31±0.24 mm compared with 2.24±0.28 mm *P*< 0.01, respectively), whereas LVIDs, LVIDd, LVs and LVd were significantly higher (*P*< 0.01). These effects were significantly reversed in the CR-SPRC and SPRC groups, which displayed higher EF, FS and LVAWs and lower LVIDs, LVIDd, LVs and LVd (*P*< 0.01 compared with the MI group). Notably, the reverse effect was also observed in the CR-SPRC group relative to the SPRC and captopril groups (Figure 6 and Table 1).

**Figure 6:**
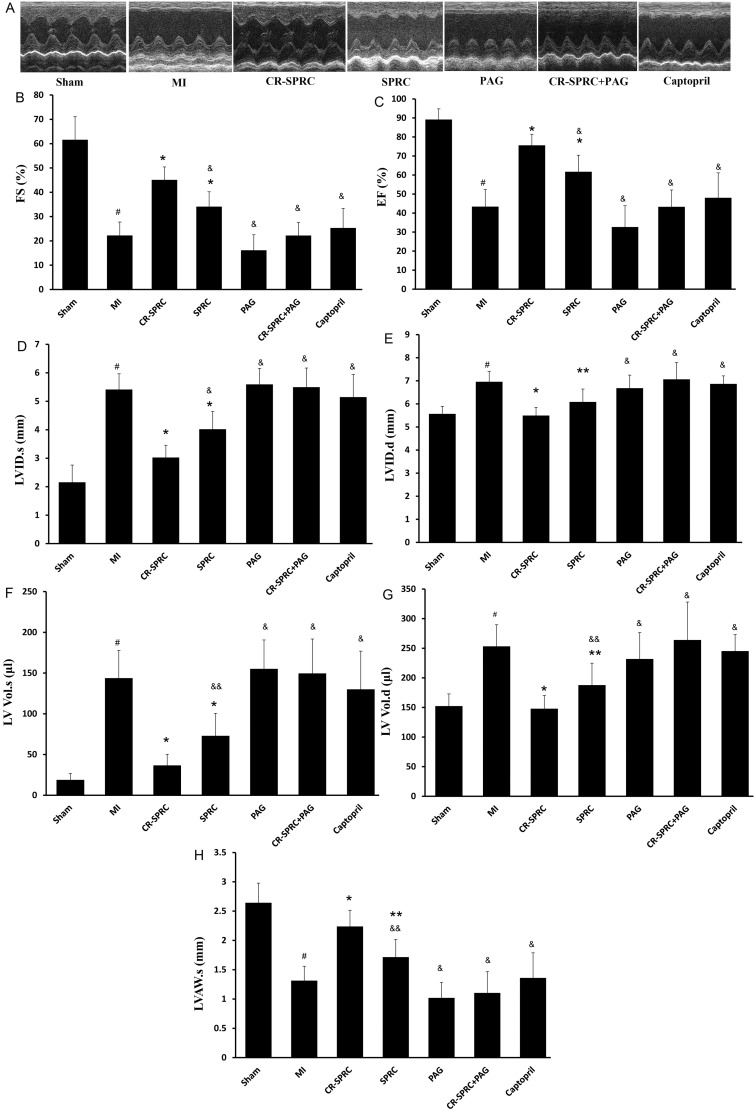
Effect of CR-SPRC on heart function Echocardiography studies. Data are presented as the mean ± S.D. (*n*=6). ^#^*P*< 0.01 compared with Sham; *P< 0.01 compared with vehicle; **P< 0.05 compared with vehicle; ^&^*P*< 0.01 compared with CR-SPRC; ^&&^*P*< 0.05 compared with CR-SPRC.

**Table 1:** Echocardiographic analysis of cardiac function

Parameter	Sham	MI	CR-SPRC	SPRC	PAG	CR-SPRC + PAG	Captopril
EF (%)	89.16±5.62	43.38±9.04*	75.62±5.82^†^	61.72±8.68^‡^^†^	32.68±11.17^‡^	43.31±8.82^‡^	48.06±13.09^‡^
FS (%)	61.62±9.45	22.26±5.49*	45.13±5.29^†^	34.10±6.11^‡^^†^	16.16±6.36^‡^	22.23±5.34^‡^	25.33±8.05^‡^
LVAWs (mm)	2.64±0.34	1.32±0.24*	2.24±0.28^†^	1.71±0.30^§^^║^	1.02±0.26^‡^	1.11±0.36^‡^	1.36±0.43^‡^
LVIDs (mm)	2.16±0.60	5.41±0.55*	3.02±0.43^†^	4.02±0.62^‡^^†^	5.6±0.56^‡^	5.5±0.67^‡^	5.15±0.80^‡^
LVIDd (mm)	5.57±0.32	6.96±0.45*	5.49±0.35^†^	6.09±0.56^║^	6.68±0.56^‡^	7.07±0.73^‡^	6.87±0.35^‡^
LV Vol.s (μl)	18.86±7.79	143.79±34.00*	36.65±13.57^†^	72.99±27.55^§^^†^	155.17±35.53^‡^	149.67±42.14^‡^	130.13±46.80^‡^
LV Vol.d (μl)	152.31±20.59	253.29±36.44*	147.91±22.40^†^	187.72±37.04^§^^║^	231.85±44.61^‡^	263.00±64.11^‡^	245.32±27.89^‡^

*P< 0.01 compared with Sham.

^†^ *P*< 0.01 compared with vehicle.

^‡^ *P*< 0.01 compared with CR-SPRC.

^§^ *P*< 0.05 compared with CR-SPRC.

^║^ *P*< 0.05 compared with vehicle.

### Pharmacokinetics of CR-SPRC

Compared with SPRC, CR-SPRC significantly reduced the *C*_max_ from 31.65±1.73 (μg/ml) to 26.10±3.68 (μg/ml) in normal rats and from 27.75±1.89 (μg/ml) to 21.02±2.37 (μg/ml) in MI model rats and it delayed the *T*_max_ from 0.8±0.3 h to 2.7±1.0 h in normal and MI rats (Figure 7 and Table 2). CR-SPRC also prolonged the mean residence time (MRT_(0–*t*))_ from 2.8±0.4 h and 2.5±0.5 h to 3.4±0.3 h and 3.5±0.3 h in normal and MI rats, respectively. The AUC_0–*t*_ was higher in CR-SPRC than in SPRC (100.7±11.1 mg/l h compared with 122.7±16.0 mg/l h in normal rats and 67.4±7.7 mg/l h compared with 102.0±14.0 mg/l h in MI rats). There was no difference in *T*_1/2_ between CR-SPRC and SPRC in normal rats, but the *T*_1/2_ of CR-SPRC was longer than that of SPRC in MI rats (1.7±0.7 h compared with 2.5±0.7 h).

**Figure 7:**
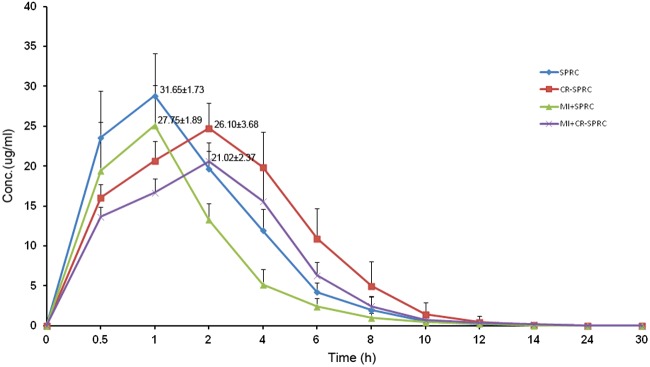
Mean plasma CR-SPRC and SPRC concentration–time curves

**Table 2:** Pharmacokinetic parameters of CR-SPRC and SPRC in normal and MI rats (mean ± S.D., *n*=6)

Parameters	SPRC	CR-SPRC	MI+SPRC	MI+CR-SPRC
*C*_max_ (μg/ml)	31.65±1.73	26.10±3.68*	27.75±1.89^†^	21.02±2.37*^‡^
*T*_max_ (h)	0.8±0.3	2.7±1.0*	0.8±0.3	2.7±1.0*
AUC_(0–*t*)_ (mg/l h)	100.7±11.1	122.7±16.0^§^	67.4±7.7^†^	102.0±14.0*^‡^
MRT _(0–*t*)_ (h)	2.8±0.4	3.4±0.3^§^	2.5±0.5	3.5±0.3*
*T*_last_ (h)	12.0±2.5	12.0±1.8	17.3±5.2^‡^	28.0±3.1*^║^

*P< 0.01 SPRC compared with CR-SPRC or MI + SPRC compared with MI + CR-SPRC.

^†^ *P*< 0.01 SPRC compared with MI + SPRC.

^‡^ *P*< 0.05 CR-SPRC compared with MI + CR-SPRC.

^§^ *P*< 0.05 SPRC compared with CR-SPRC or MI + SPRC compared with MI + CR-SPRC.

^║^ *P*< 0.01 CR-SPRC compared with MI + CR-SPRC.

## DISCUSSION

Acute MI often occurs suddenly; however, current therapies are not always quickly available, leading to an unfavourable outcome. Therefore, the best strategy for the management and prevention of MI is the administration of preventive treatment to high-risk patients to reduce the occurrence and mitigate the severity of MI. Controlled release formulations have the ability to maintain an effective drug concentration that could decrease the frequency of administration and reduce the required dosage of drugs, while minimizing side effects and improving patient compliance. These characteristics of controlled release formulations have made them an attractive option to modify the pharmacokinetic properties of SPRC for prevention of MI.

In the present study, we compared the cardioprotective effects of CR-SPRC and SPRC in an MI model and demonstrated that CR-SPRC had better cardioprotective effects than SPRC. Consistent with previous studies, CR-SPRC induced higher CSE protein expression and elevated plasma H_2_S. Importantly, CR-SPRC was able to maintain CSE in myocardial tissue and hydrogen sulfide levels in plasma at a higher level than SPRC.

There is a link among increased inflammatory reaction, oxidative stress and aggravated myocardial damage in MI. MI involves the activation of multiple stress signalling processes, such as oxidative stress, that generate reactive oxygen species (ROS), leading to the expression of inflammatory mediators, including TNF-α, which induces Iκ-Bα degradation and NF-κβ phosphorylation. This cascade results in vigorous inflammatory reactions that promote myocardial apoptosis, cell death, ventricular remodelling and cardiac dysfunction. Therefore, preventing TNF-α-induced Iκ-Bα degradation or suppressing TNF-α production to inhibit NF-κβ p65 subunit phosphorylation could preserve myocardial function [23,24]. Our findings are consistent with a study by Pan et al. [4], which reported that SPRC attenuated the LPS-induced inflammatory response in H9c2 cells by inhibiting TNF-α expression, preventing NF-κB activation and reducing Iκ-Bα degradation. By modulating the CSE/H_2_S pathway, CR-SPRC treatment inhibited inflammatory mediators of TNF-α expression and inhibited Iκ-Bα degradation and NF-κβ p65 subunit phosphorylation. Moreover, we confirmed that CR-SPRC provided a better anti-inflammatory effect than SPRC.

Recent controversy over the role of H_2_S in systemic inflammatory diseases has dogged molecular pathology. In earlier studies, H_2_S was demonstrated to play a pro-inflammatory role in systemic inflammation [25–27], whereas recent evidence suggests that H_2_S may exert anti-inflammatory effects via multiple mechanisms [28]. These controversial may have resulted from differences in the concentration of H_2_S, which is a small gaseous molecule that can be easily released and diffused, making it difficult to maintain an effective concentration. Our study suggests that by maintaining the release of endogenous hydrogen sulfide, CR-SPRC could provide a promising approach for the treatment of inflammatory vascular diseases.

Oxidative stress, including elevated ROS levels in the myocardium following infarction, plays a critical role in cardiac myocyte death and loss of cardiac function [29,30]. SOD and CAT activity and GSH and MDA levels are the principal patho-physiological parameters that determine antioxidant capability. Wang et al. [5] also reported that SPRC enhanced the cellular antioxidant defence in MI rats by maintaining CAT and SOD activity and GSH plasma levels, whereas decreasing MDA levels. In this study, which used a lower dose than the Wang study (30 mg/kg compared with 50 mg/kg), CR-SPRC prevented MI-triggered oxidative stress by preserving CAT and SOD activity and GSH levels, resulting in lower MDA levels. Furthermore, the MDA level of the CR-SPRC-treated rats was lower than that of SPRC-treated rats, indicating that CR-SPRC has a more favourable antioxidant effect than SPRC. More importantly, the SOD activity and GSH levels were significantly higher in the CR-SPRC plus PAG treatment group than in the MI group, suggesting that CR-SPRC could maintain long-term efficacy even in the presence of PAG (a CSE inhibitor). Moreover, oxidative stress is also a major source of NF-κB activation, and the attenuation of oxidative stress by CR-SPRC may contribute to suppression of NF-κβ activation [31].

In this study, we demonstrated that the anti-inflammatory and antioxidative effects of CR-SPRC provide preferable cardioprotective effects relative to SPRC. CR-SPRC reduced infarct size and mortality, limited cardiac remodelling and improved cardiac function, as reflected by higher EF, FS and LVAWs and lower LVIDs, LVIDd, LVs and LVd. These indicators are critical for the prognostic outcome of MI, and their associated effects were especially noticeable in response to CR-SPRC compared with SPRC and captopril, with lower CK and LDH levels, reduced mortality and infarct size and better cardiac function, in addition to higher EF, FS and LVAWs and lower LVIDs, LVIDd, LVs and LVd.

H_2_S is synthesized endogenously in various mammalian tissues by two pyridoxal-5′-phosphate-dependent enzymes cystathionine β-synthase (CBS) and CSE, each of which utilize L-cysteine as substrate. CBS is the main producer of H_2_S in the central nervous system, whereas CSE is a major H_2_S-producing enzyme in the cardiovascular system. A recent study also discovered another enzyme 3-mercaptopyruvate sulfur transferase (3-MST) that can generate endogenous H_2_S. 3-MST is located in the liver, kidney, heart, lung, thymus, testis, thoracic aorta and brain. Unlike CBS and CSE, 3-MST uses 3-mercaptopyruvate, which is a metabolite of cysteine and keto acids catalysed by cysteine aminotransferase, as a substrate to form H_2_S [9,19]. Due to structure similar to cysteine, SPRC could increase the expression of CSE and H_2_S production. Based on these data above, in this study, we verified that CR-SPRC acts through the CSE/H_2_S-mediated pathway by administering PAG (an irreversible CSE inhibitor), which has been used in previous studies to test the biological effect of inhibiting endogenous H_2_S production. The PAG groups had the lowest levels of CSE, H_2_S, CAT, SOD and GSH, and the highest levels of CK, LDH and MDA, which were associated with the largest infarct size and lowest cardiac function. Moreover, CSE inhibitor decreased the expression of CSE when coadministered with CR-SPRC and abolished the cardioprotective effects of CR-SPRC. These data further confirm that CR-SPRC increased the expression of CSE and the CSE/H_2_S pathway plays an important role in CR-SPRC-induced cardioprotection.

To explore the mechanism by which CR-SPRC exerts increased cardioprotective relative to SPRC, we used mixed-mode, reversed-phase and cation-exchange HPLC–MS/MS to determine SPRC plasma concentrations. CR-SPRC maintained stable levels of SPRC by reducing the concentration peak, delaying the time to reach the peak concentration and prolonging the mean residence time, which improved drug bioavailability. By prolonging the release of endogenous hydrogen sulfide or the SPRC plasma level and reducing the concentration peak of SPRC, CR-SPRC could prevent hydrogen sulfide toxicity.

In summary, CR-SPRC displayed a better cardioprotective effect than SPRC, which is consistent with a prolonged maintenance of the endogenous H_2_S concentration. Our study confirms that CR-SPRC is stable, effective and is thus an alternative candidate for hydrogen sulfide-mediated long-term prevention of cardiovascular diseases.

## Abbreviations

CAT: catalase
CBS: cystathionine β-synthase
CK: creatine kinase
CMC-Na: sodium carboxymethylcellulose
CR-SPRC: controlled release *S*-propargyl-cysteine
CSE: cystathionine-γ-lyase
EF: ejection fraction
FS: fractional shortening
GSH: glutathione
LDH: lactate dehydrogenase
LVd: left ventricular volume in diastole
LVs: left ventricular volume in systole
LVAWs: left ventricular anterior wall in systole
LVIDd: left ventricular internal dimension in diastole
LVIDs: left ventricular internal dimension in systole
MDA: malondialdehyde
MI: myocardial infarction
3-MST: 3-mercaptopyruvate sulfur transferase
PAG: propagylglycine
SOD: superoxide dismutase
SPRC: *S*-propargyl-cysteine
TTC: 2,3,5-triphenyltetrazolium chloride
